# Supplementary material for: Pitfalls in time-to-event analysis of registry data: a tutorial based on simulated and real cases
Source: Front Epidemiol. 2024 Jul 11;4:1386922. doi: 10.3389/fepid.2024.1386922 (PMC11345615; doi:10.3389/fepid.2024.1386922)

**List of Supplementary Material**

Supplementary data 1: Construction of the Kaplan-Meier estimator.

Supplementary data 2: True cumulative incidence function in a competing risk situation, using two Weibull distributions for the simulation.

Supplementary figure 1: Survival probabilities with different starting points, with 95% confidence interval. (a) Post-diagnosis survival for patients with CVID, by age at diagnosis. (b) Overall survival from birth, by PID category.

Supplementary figure 2: The probabilities of developing a first cancer and its competing event, according to the main PID categories, with 95% confidence interval. (a) the probability of developing a first cancer, according to the main PID categories. (b) the probability of death or receiving curative therapy (the competing risk), whichever comes first and according to the main PID categories.

**Appendix**

https://github.com/Malligon/Pitfalls-in-Time-to-Event-Analysis-for-Registry-Data

Supplementary data 1: Construction of the Kaplan-Meier estimator

In this paragraph, we provide the mathematical arguments for the calculation of the Kaplan-Meier estimator. Starting from the grid of time points t_1_, …, t_K_ consisting of all the uncensored times, the estimator is computed recursively. For a given t_k_, the Kaplan-Meier estimator is equal to its value at the previous time point t_(k-1)_ times (1-d_k_/R_k_), where d_k_ is the number of uncensored events that occurred at time t_k_, and R_k_ is the number of “at risk” individuals at time t_k_ (defined as the number of individuals that have not yet experienced the event of interest or who have not yet been censored). In this formula, the estimator is initialized at the value 1 for time equal to 0. In survival analysis, the notion of individuals being “at risk” at a given time is essential. It is only through this risk set that censoring is accounted for, and it is crucial that this set does not include periods of time during which the event of interest cannot occur. The d_k_/R_k_ ratio is called the hazard rate or hazard risk estimator and represents the estimation for the risk of experiencing the event of interest at time t_k_, given that this event has not yet occurred.

In Section 3A and Figure 1, we showed that the naïve approach (which does not take into account right-censored observations) is a biased estimator of the survival function as compared to the Kaplan-Meier estimator. By way of an example, one can consider a study in which the event of interest is death: including only observed deaths in the analysis (and thus ignoring censored data due to the end of study or drop-out) will result in underestimation of the survival function. This is because at all time points, the risk set needs to include the censored observations that have not yet occurred. Whereas the number of observed events of interest is the same (d_k_ in the Kaplan-Meier estimator), the number of individuals at risk (R_k_ in the Kaplan-Meier estimator) should be increased.

Supplementary data 2: True cumulative incidence function in a competing risk situation, using two Weibull distributions for the simulation

The two competing risks are simulated from two Weibull distributions with k1 and k2 the shape parameters and λ1 and λ2 the scale parameters. We assume k=k_1_=k_2_ and in the following we note $t^v$ the minimum between $t$ and $v$ and $f_{T_{1}}$represents the density of the first Weibull distribution, $f_{T_{2}}$the distribution of the second Weibull distribution. We have:

$$P\left( T_{1}\leq t \cap T_{1}\leq T_{2} \right) =E(1_{T_{1}\leq t \& T_{1}\leq T_{2}})$$

$$= \iint1_{u\leq t}1_{u\leq v}f_{T_{1}}\left( u \right)f_{T_{2}}\left( v \right)dudv$$

$$= \int_{0}^{+\infty} \int_{0}^{t^v} f_{T_{1}}\left( u \right){du f}_{T_{2}}\left( v \right)dv$$

$$= \int_{0}^{+\infty} \left[ -e^{-(\frac{u}{\lambda_{1}})^{k}} \right]_{0}^{t^v}f_{T_{2}}\left( v \right)dv$$

$$=1-\int_{0}^{+\infty} \frac{k_{2}}{\lambda_{2}}({\frac{v}{\lambda_{2}})}^{k-1}e^{-({\frac{v}{\lambda_{2}})}^{k}}e^{-(\frac{t^v}{\lambda_{1}})^{k}}dv$$

$$=1-(\int_{0}^{t} \frac{k_{2}}{\lambda_{2}}({\frac{v}{\lambda_{2}})}^{k-1}e^{-({\frac{v}{\lambda_{2}})}^{k}}e^{-(\frac{v}{\lambda_{1}})^{k}}dv+ \int_{t}^{+\infty} \frac{k}{\lambda_{2}}({\frac{v}{\lambda_{2}})}^{k-1}e^{-({\frac{v}{\lambda_{2}})}^{k}}e^{-(\frac{t}{\lambda_{1}})^{k}}dv)$$

First,

$$\int_{t}^{+\infty} \frac{k}{\lambda_{2}}({\frac{v}{\lambda_{2}})}^{k-1}e^{-({\frac{v}{\lambda_{2}})}^{k}}e^{-(\frac{t}{\lambda_{1}})^{k}}dv = e^{-(\frac{t}{\lambda_{1}})^{k}}\left[ -e^{-(\frac{v}{\lambda_{2}})^{k}} \right]_{t}^{+\infty}$$

$$= e^{-(\frac{t}{\lambda_{1}})^{k}-(\frac{t}{\lambda_{2}})^{k}}$$

Then, for the calculation of $\int_{0}^{t} \frac{k_{2}}{\lambda_{2}}({\frac{v}{\lambda_{2}})}^{k-1}e^{-({\frac{v}{\lambda_{2}})}^{k}}e^{-(\frac{v}{\lambda_{1}})^{k}}dv,$ we set $u(v)=-(\frac{v}{\lambda_{1}})^{k}-({\frac{v}{\lambda_{2}})}^{k}$ and $x=$ $\frac{\lambda_{1}}{\lambda_{2}}$ such that

$u^{'}\left( v \right)=$ $-\frac{k}{\lambda_{1}}(\frac{v}{\lambda_{1}})^{k-1}-\frac{k}{\lambda_{2}}(\frac{v}{\lambda_{2}})^{k-1}$

$$u^{'}(v)= -\frac{k}{{x\lambda}_{2}}(\frac{v}{{x\lambda}_{2}})^{k-1}-\frac{k}{\lambda_{2}}(\frac{v}{\lambda_{2}})^{k-1}$$

$$u^{'}(v)=-\frac{k}{\lambda_{2}}(\frac{1}{x}(\frac{v}{{x\lambda}_{2}})^{k-1}+ (\frac{v}{\lambda_{2}})^{k-1})$$

$$u^{'}(v)=-\frac{k}{\lambda_{2}}(\frac{v}{\lambda_{2}})^{k-1}(\frac{1}{x^{k}}+1)$$

We can now write:

$$\int_{0}^{t} \frac{k}{\lambda_{2}}({\frac{v}{\lambda_{2}})}^{k-1}e^{-({\frac{v}{\lambda_{2}})}^{k}}e^{-(\frac{v}{\lambda_{1}})^{k}}dv= \int_{0}^{t} -\frac{1}{(\frac{1}{x^{k}}+1)}u'(v)e^{u(v)}dv$$

$$= -\frac{1}{\left( \frac{1}{x^{k}}+1 \right)}\int_{0}^{t} u^{'}(v)e^{u(v)}dv$$

$$=-\frac{1}{\left( \frac{1}{x^{k}}+1 \right)} \left[ e^{-(\frac{v}{\lambda_{1}})^{k}-(\frac{v}{\lambda_{2}})^{k}} \right]_{0}^{t}$$

$$= -\frac{1}{\left( \frac{1}{x^{k}}+1 \right)} (e^{-(\frac{t}{\lambda_{1}})^{k}-(\frac{t}{\lambda_{2}})^{k}}-1)$$

$$= -\frac{1}{\left( \frac{{\lambda_{2}}^{k}}{{\lambda_{1}}^{k}}+1 \right)} (e^{-(\frac{t}{\lambda_{1}})^{k}-(\frac{t}{\lambda_{2}})^{k}}-1)$$

Finally, gathering all the terms we obtain:

$$P\left( T_{1}\leq t \cap T_{1}\leq T_{2} \right) =1-(e^{-(\frac{t}{\lambda_{1}})^{k}-(\frac{t}{\lambda_{2}})^{k}}+ -\frac{1}{\left( \frac{{\lambda_{2}}^{k}}{{\lambda_{1}}^{k}}+1 \right)} \left( e^{-(\frac{t}{\lambda_{1}})^{k}-(\frac{t}{\lambda_{2}})^{k}}-1 \right))$$

$$P\left( T_{1}\leq t \cap T_{1}\leq T_{2} \right) =1-({\frac{1}{\left( \frac{{\lambda_{2}}^{k}}{{\lambda_{1}}^{k}}+1 \right)}+e}^{-(\frac{t}{\lambda_{1}})^{k}-(\frac{t}{\lambda_{2}})^{k}}\left( -\frac{1}{\left( \frac{{\lambda_{2}}^{k}}{{\lambda_{1}}^{k}}+1 \right)}+1 \right))$$

$$=1-(\frac{{\lambda_{1}}^{k}}{{\lambda_{1}}^{k}+{\lambda_{2}}^{k}}+ e^{-\left( \frac{{\lambda_{1}}^{k}+{\lambda_{2}}^{k}}{{\lambda_{1}}^{k}{\lambda_{2}}^{k}} \right)t^{k}}\left( 1-\frac{{\lambda_{1}}^{k}}{{\lambda_{1}}^{k}+{\lambda_{2}}^{k}} \right))$$

Supplementary figure 1 (a): Post-diagnosis survival for patients with CVID, by age at diagnosis.


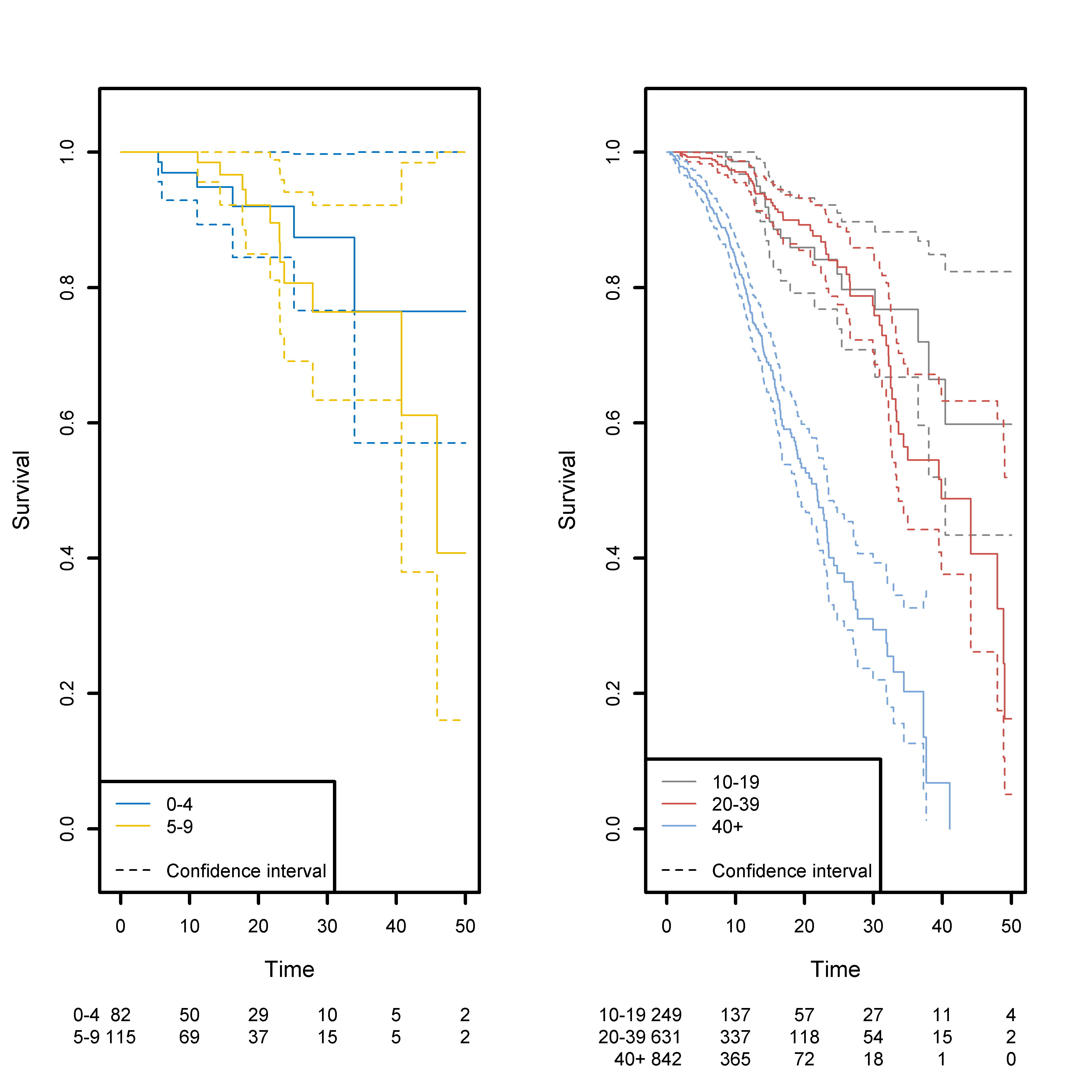


Supplementary figure 1 (b): Overall survival from birth, by PID category.


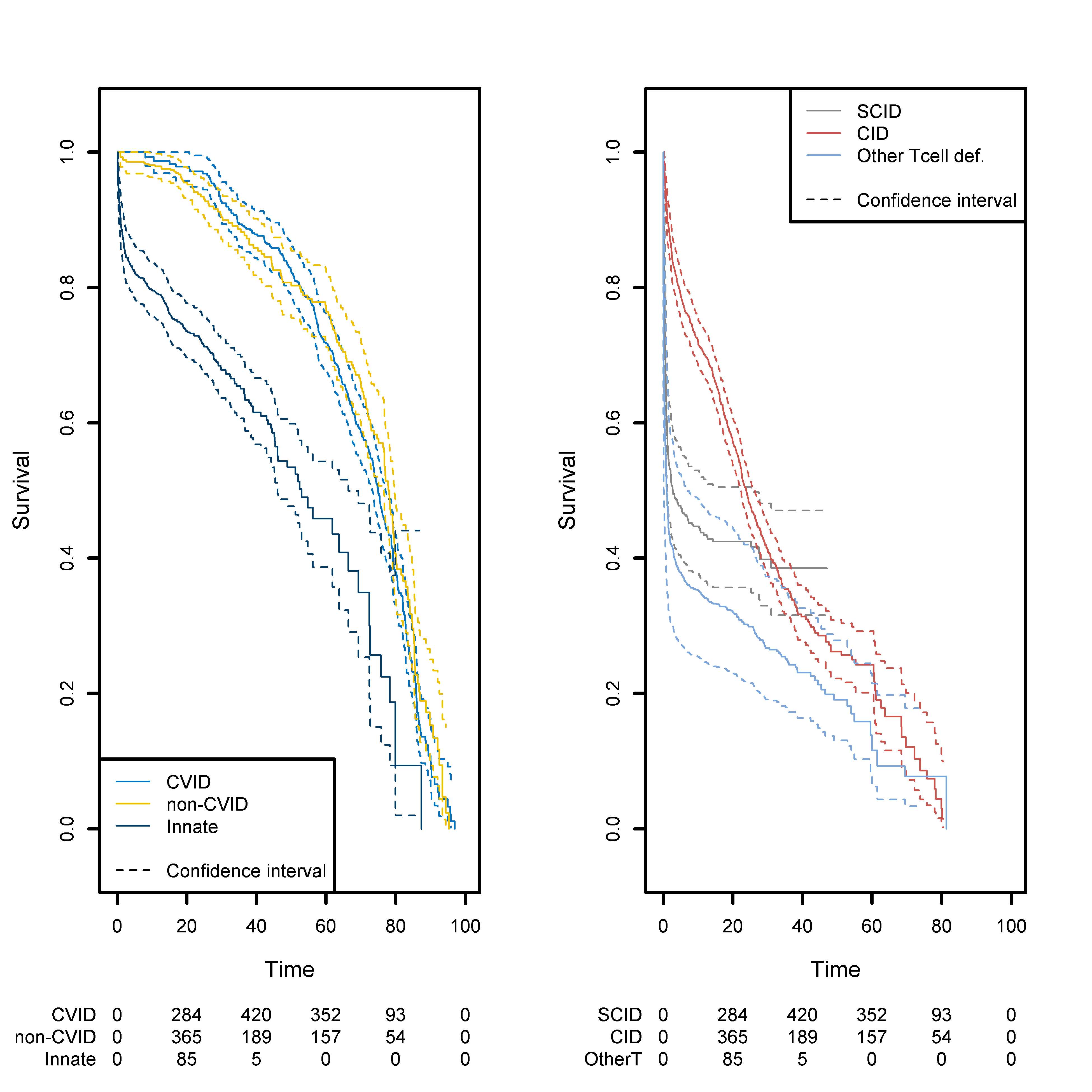


Supplementary figure 2 (a): The probability of developing a first cancer, according to the main PID categories.


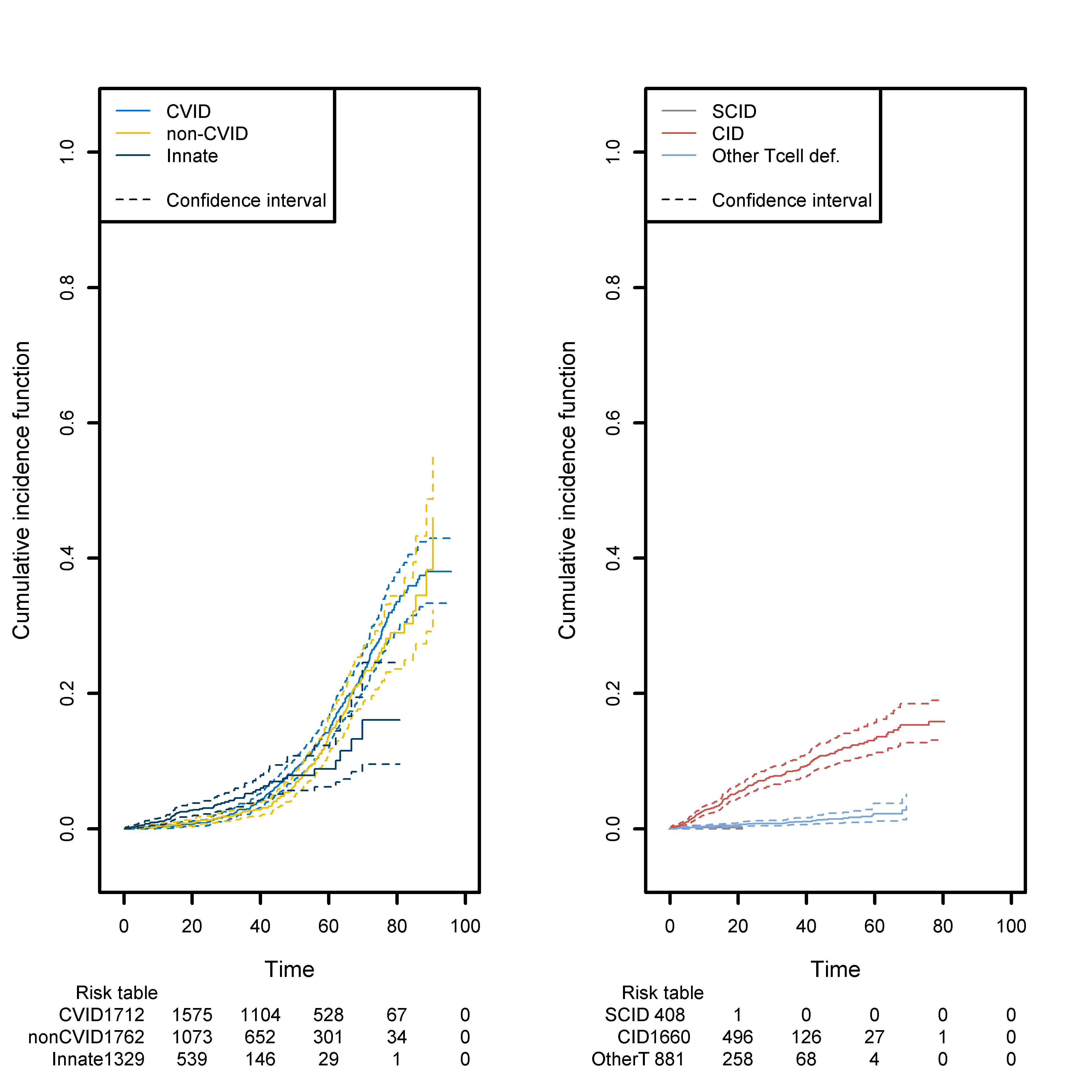


Supplementary figure 2 (b): The probability of death or receiving curative therapy (the competing risk), whichever comes first and according to the main PID categories.


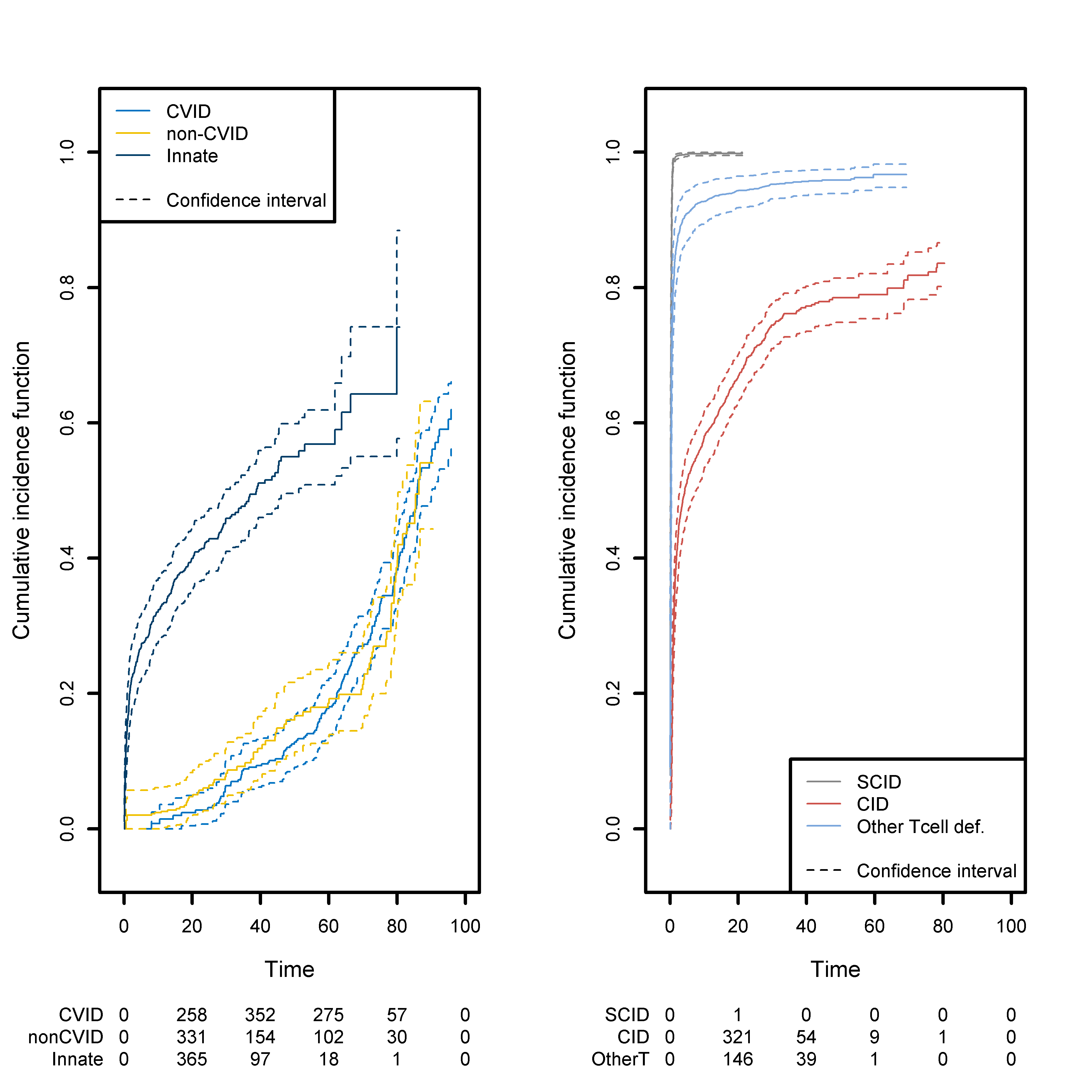

Supplement: Supplementary file 1 [file Datasheet1.docx]
